# A trait profile of top and middle managers

**DOI:** 10.3389/fpsyg.2015.01694

**Published:** 2015-11-04

**Authors:** Anna K. Baczyńska, Tomasz Rowiński

**Affiliations:** ^1^Department of Management, Kozminski UniversityWarsaw, Poland; ^2^Cardinal Stefan Wyszyński University in WarsawWarsaw, Poland

**Keywords:** general mental ability, top and middle managers, profile, personality, emotional intelligence

## Introduction

Although trait leadership theory has been widely criticized by scholars over the past century (Stogdill, 1948; Mann, 1959) employers always want to know what kind of abilities and psychological traits distinguish their future employees. The trait approach plays an important part in human resource management practices for selecting, developing and planning the career paths of managers (Armstrong, 2010). Interestingly, Zaccaro (2007) points out that even Stogdill (1948) review, although cited as evidence against leader traits, contains conclusions suggesting that individual differences can still predict leader effectiveness. Trait leadership is defined as integrated patterns of personal characteristics that reflect a range of individual differences and foster consistent leader effectiveness across a variety of group and organizational situations (Zaccaro et al., 2004).

Top and middle level managers play different roles in organizations and therefore require different skills (Koźmiński, 2011, 2013; Griffin, 2013). In many studies, top managers are shown to differ from middle managers in terms of (1) analytical mental ability (Jensen, 1998; Schmidt and Hunter, 1998; Judge et al., 2004; Sternberg, 2007; Caruso et al., 2013); (2) emotional intelligence (Miller, 1999; Barling et al., 2000; Palmer et al., 2001; Gardner and Stough, 2002; Goleman et al., 2002; Goleman, 2006; Boyatzis, 2011; Caruso et al., 2013); and (3) personality (Judge et al., 2002; Hogan and Holland, 2003; Bono and Judge, 2004). Other results underline that mental ability and personality together play a significant role in management (Lepine et al., 2000).

## Materials and methods

### Participants

Our research was conducted at the beginning in 2015 year. The study sample consisted of 233 managers (aged 27–54 years, *M* = 35.26; *SD* = 4.77), formed of two groups: (1) managers currently undertaking MBA studies who hold positions of Chairman, Managing Director, Departmental Director or Regional Director (*F* = 18, *M* = 60; *M*_*age*_ = 34.90; *SD*_*age*_ = 4.97) in organizations, and (2) participants of post-graduate managerial courses at Koźmiński University in Warsaw, Poland (*F* = 59, *M* = 84; *M*_*age*_ = 35.98; *SD*_*age*_ = 4.27) who hold positions such as departmental or section manager, or team leader. The managers were selected from fields such as “fast-moving consumer goods,” automotives, pharmaceuticals and IT, worked a minimum three years in their position, and received the highest scores in performance appraisals at work. The participants (*N* = 233) took part in the study voluntarily. During the first session of Career Development module, each participant received three scales: Raven's Progressive Matrices (RPM), the Emotional Intelligence Questionnaire (EIQ) and the Circumplex of Personality Metatraits Portrait (CPM-P). They fulfilled RPM in the classroom, the next ones we collected in month. Participants received their results on the individual feedback session. In further stages of our analysis we included only participants who completed the tests without missing responses. There were 220 participants who completed RPM (*M* = 143; *F* = 77; 94.4% response rate), 176 who completed EIQ (*F* = 63; *M* = 113; 75.5% response rate), and 190 who completed CPM-P (*F* = 68; *M* = 122; 81.5% response rate).

### Materials

#### Raven's progressive matrices

This scale is designed to measure general mental ability (general intelligence). We used the standard progressive version to investigate the level of analytical ability of managers. This is a popular tool of measurement for general mental ability and its reliability (Cronbach's *alpha* is above 0.80) and validity have been empirically proven in many studies (Raven, 2000; Shamosh and Gray, 2007; Harrison et al., 2015; Little and McDaniel, 2015).

#### Emotional intelligence questionnaire

This scale was developed by Jaworowska and Matczak (2005). The tool is based on the Salovey and Mayer (1989) approach of emotional intelligence and consists of a scale of four theoretical concepts: (1) *Acceptance*—the measured ability to accept, express and use emotions in action, (2) *Emotional Understanding*—the ability to understand one's own emotions, (3) *Control*—the ability to control one's own emotions, (4) *Empathy*—the ability to understand and recognize emotions in other people. Reliability measured using Cronbach's *alpha* is good and is above 0.76 for all scales. Participants indicate their level of agreement with statements on a 5-point Likert-type scale from 1—*I strongly disagree* to 5—*I strongly agree*. Sample statements are: *When someone is angry, I can usually feel it; Often I don‘t know if someone really likes me or is just acting*.

#### Circumplex of personality metatraits portrait

In order to measure personality we used a scale which investigates the personality dimension in a circumplex model. This is reliable (Cronbach's *alpha* for all scales is above 0.70) and validates 54 items, which are scored on a 7-point Likert-type scale ranging from 1—*not similar to me at all* to 7—*very similar to me*. Sample items are*: He/She feels good both in the company of others as well as by himself/herself. In every situation retains inner harmony and serenity. He/She feels happy and optimistic about the future; He/She likes to engage in new initiatives. He/She quickly adapts to new situations*.

According to the authors Strus et al. (2014), a metatrait can be understood as a personality dimension which differentiates people in thinking, behavior and emotions. The model consists of eight measurements: (1) *Plasticity* (0° in the circle) is linked to a tendency toward exploration of the environment, cognitive, and behavioral openness to change, engagement with new experiences, an individual tendency to widen one's horizons, (2) *Integration* (45° in the circle) is a positive, pro-social attitude to people, a balance between work and family, the successful realization of life goals, (3) *Stability* (90° in the circle) signifies stable functioning in emotional, motivational, and social spheres, (4) *Self-Restraint* (135° in the circle) represents low emotionality, unwillingness to show emotions, strong control of one's behavior and conformism, (5) *Passiveness* (180° in the circle) constitutes cognitive and behavioral passivity, apathy, and submissiveness, (6) *Disharmony* (225° in the circle) represents withdrawal from social and professional activity, a distrustful attitude, distance from others, a tendency to view events and the world pessimistically, (7) *Disinhibition* (270° in the circle) indicates a tendency toward emotional instability, low resistance to frustration, aggression, and antagonism toward people and governing rules, (8) *Sensation-Seeking* (315° in the circle) signifies impulsiveness, emotional liability, sensation-seeking, a desire to dominate and expansiveness in interpersonal relations.

### Dataset description

The managers dataset, called *Data Managers*, is deposited at https://www.dropbox.com/s/vxlrazavrai4lfx/Data_Managers.sav?dl=0 and has SPSS 22 format. The variables are described within.

## Funding

This research was partially supported by grants DEC- 2014/15/B/HS4/04428 from the National Science Centre, Poland.

### Conflict of interest statement

The authors declare that the research was conducted in the absence of any commercial or financial relationships that could be construed as a potential conflict of interest.
